# Insights from a national survey into why substance abuse treatment units add prevention and outreach services

**DOI:** 10.1186/1747-597X-1-21

**Published:** 2006-08-03

**Authors:** Rebecca Wells, Christy Harris Lemak, Thomas A D'Aunno

**Affiliations:** 1 Department of Health Policy and Administration, School of Public Health, University of North Carolina, Chapel Hill, North Carolina, USA; 2 Department of Health Services Research, Management and Policy, College of Public Health and Health Professions, University of Florida, Gainesville, Florida, USA; 3 INSEAD, Fontainebleu Cedex, France

## Abstract

**Background:**

Previous studies have found that even limited prevention-related interventions can affect health behaviors such as substance use and risky sex. Substance abuse treatment providers are ideal candidates to provide these services, but typically have little or no financial incentive to do so. The purpose of this study was therefore to explore why some substance abuse treatment units have added new prevention and outreach services. Based on an ecological framework of organizational strategy, three categories of predictors were tested: (1) environmental, (2) unit-level, and (3) unit leadership.

**Results:**

A lagged cross-sectional logistic model of 450 outpatient substance abuse treatment units revealed that local per capita income, mental health center affiliation, and clinical supervisors' graduate degrees were positively associated with likelihood of adding prevention-related education and outreach services. Managed care contracts and methadone treatment were *negatively *associated with addition of these services. No hospital-affiliated agencies added prevention and outreach services during the study period.

**Conclusion:**

Findings supported the study's ecological perspective on organizational strategy, with factors at environmental, unit, and unit leadership levels associated with additions of prevention and outreach services. Among the significant predictors, ties to managed care payers and unit leadership graduate education emerge as potential leverage points for public policy. In the current sample, units with managed care contracts were less likely to add prevention and outreach services. This is not surprising, given managed care's emphasis on cost control. However, the association with this payment source suggests that public managed care programs might affects prevention and outreach differently through revised incentives. Specifically, government payers could explicitly compensate substance abuse treatment units in managed care contracts for prevention and outreach. The effects of supervisor graduate education on likelihood of adding new prevention and outreach programs suggests that leaders' education can affect organizational strategy. Foundation and government officials may encourage prevention and outreach by funding curricular enhancements to graduate degree programs demonstrating the importance of public goods.

Overall, these findings suggest that both money and professional education affect substance abuse treatment unit additions of prevention and outreach services, as well as other factors less amenable to policy intervention.

## Background

As policy makers grapple with the persistent social and economic costs of substance abuse and other risky behaviors, the need for prevention remains starkly evident. Studies have found that even brief, informational interventions can lead to significant reductions in risky behaviors [1-4]. Given the enormous human and financial costs involved, the need for such preventive efforts is obvious. A critical question is who will provide them.

The current study addresses this question in the context of substance abuse prevention, specifically asking: "What factors make it more likely that substance abuse treatment units will add new community prevention, education, and outreach services?" The current inquiry focuses on outpatient substance abuse treatment providers, which provide the majority of substance abuse treatment in the United States [5]. These units may be self standing or based in hospitals, mental health centers, or other facilities. Thus, like many health and human service providers, substance abuse treatment units are often organizations embedded in larger organizations.

This study employs The Washington Circle's definition of "prevention/education" as "delivery system activities designed to raise the general awareness of substance abuse as a major debilitating disorder affecting individuals, families, and the greater society... [and] ... those activities designed to target high-risk individuals and groups for more focused interventions" [[6], p. 638]. The Washington Circle thus highlights prevention/education as a domain within the continuum of substance abuse treatment.

The focus here is on decisions by substance abuse treatment units to add new prevention-related education and outreach services. These included programs related to substance abuse as well as HIV/AIDS, a continuing epidemic whose spread is closely linked to substance use. The vast majority of outpatient substance abuse treatment centers already provide some type of community outreach or prevention services [7]. The current investigation therefore focused on factors related to whether units extended themselves in this vital, yet under-reimbursed, area.

This inquiry was premised on two key assumptions. The first was that substance abuse treatment units were ideal candidates to provide prevention-related education and outreach because they had staff members who were knowledgeable about how to help people avoid risky behaviors as well as about local human service resources. The second was that prevention and outreach were "public goods" in that society clearly benefited from these efforts. In the United States, however, third party reimbursement for prevention services was generally low or nonexistent [8,9]. Thus, what was good for society could be financially risky for organizations. It was therefore not sufficient to use financial incentives alone when examining why substance abuse treatment units would add new prevention and outreach services.

### Conceptual model and predictions

In addition to the assumptions noted above, the investigation outlined here builds on two conceptual perspectives. First, the outcome is viewed as part of organizational strategy. That is, whether the additions of prevention and outreach emerge from formal, top-down planning or result from incremental operational decisions, such diversifications entail resource commitments and affect the ways providers serve their markets [10].

Second, building on previous analyses from the same national survey utilized here, the authors employ an ecological perspective on factors potentially affecting organizational strategy. Previous analyses of these data have revealed environmental, organizational, and facility leadership factors to be associated with the likelihood of forming cooperative relationships with other agencies [11] as well as with facility survival [12]. The current investigation extends this research program, this time with respect to a societally vital type of service diversification.

Traditionally, different "schools" of strategic thought have emphasized environmental, organizational, and leadership factors respectively [13]. There is utility to focusing deeply on one level of any given organizational phenomenon at a time. This study, however, seeks to reveal how well *different *levels of units' ecologies affect the establishment of new prevention and outreach services. Previous research indicates that factors that may affect strategic change include market conditions [14] and relationships with other actors [15], organizational capabilities and norms [16], and those of the individuals leading them [17]. Thus, the current inquiry included local market conditions and relationships with other organizations at the environmental level; attributes of the units themselves; and, finally, the attributes of administrative and clinical leadership.

### Predictions about environmental factors

At the environmental level, two market and three inter-organizational factors appeared potentially relevant to additions of prevention and outreach services. The first market factor expected to facilitate such additions was local affluence, as resource availability often supports more generous public goods (e.g., affluent areas have better municipal services). The second potentially relevant market factor was competition. Previous evidence from hospitals indicates that competition encourages broader service offerings [18,19]. This is consistent with the tendency for organizations to respond to environmental pressures with strategies intended to reduce reliance on any one source of income [20]. The question was whether this would apply to service that generally cost money but did not generate revenue. If organizations generally add services to respond to competition in ways to diversify their revenue base, treatment units facing greater competition might *refrain *from adding prevention and outreach.

Relationships with other organizations are another vital element of unit environments. Two key types of external partners are payers and parent organizations. For behavioral health care providers, an increasingly important type of payer is managed care. There is evidence that substance abuse units involved with managed care reduce some services [21], although there have not been similar findings from other health care sectors [22,23]. Thus, no specific prediction was appropriate about the effect of managed care on likelihood of adding prevention or outreach. Because of the now salient role of managed care in behavioral health [24], however, this factor was included in the predictive model.

Organizations may also be based in parent organizations that affect their decisions about services. One major motivation for diversification is to complement existing services. Hospitals have generally not historically emphasized prevention and outreach. Faced with increasing pressure to become more prevention and community-oriented, they may have encouraged units to add such services. Conversely, units based in mental health centers might be less likely to add prevention and outreach because these services are generally part of the parent organizations' existing portfolios.

The catch is that strategic theory assumes profit motivation, which does not apply to public goods such as prevention and outreach services. Another potentially applicable perspective is institutional theory [25]. Institutional theory predicts that belief systems affect strategic decisions, irrespective of their financial implications. The result is an opposite set of predictions. In other words, because hospitals are culturally oriented toward remediation rather than prevention, institutional theory implies that their leadership would not emphasize prevention-related outreach. Conversely, mental health center administrators, having a more public health ethos, would be more supportive of adding prevention and outreach services.

The evidence to date does not suggest such differential predictions. Instead, it appears that inter-organizational relationships are generally conducive to prevention activities. Previous research has found agency relationships with other human service organizations to be positively related to AIDS education for current clients [26] and hospitals' network and alliance participation to be positively associated with more prevention services [23]. On balance, however, because of limitations to the relevance of strategic theory to public goods, the authors relied on institutional theory rather than on empirical findings to date. Thus, based on the assumption that key partners' belief systems affect strategic decisions, the expectation was that units based in hospitals would be less likely to add prevention and outreach services and those based in mental health clinics would be more likely to add these services.

These predictions can be summarized as follows:

H1. Substance abuse treatment units in higher income areas will be more likely to add prevention and outreach services.

H2. Units whose directors perceive more competition will be less likely to add prevention and outreach services.

[No prediction made about managed care, but included in model due to relevance of payment mechanisms.]

H3. Hospital-based units will be less likely to add prevention and outreach services.

H4. Units based in mental health centers will be more likely to add prevention and outreach services.

### Predictions about substance abuse treatment unit-level factors

The predictions outlined above have drawn on management theories emphasizing organizations' relationships with their environments. At the same time, however, attributes of organizations themselves may affect what strategies they pursue and how. In the context of decisions about new service offerings, the financial structures, existing services, and community interfaces of the units themselves may also be relevant. Previous studies indicated that for-profits would be less likely than other facilities to add prevention or outreach [23], but did not support any predictions about significantly different likelihoods between public and nonprofit facilities [27]. Previous findings indicated that methadone units would be more likely than others to add these services [27]. All the units in the current study were outpatient, and thus drew on local clientele. However, methadone units differ from other treatment facilities in having more functional (physiologically stabilized) clients who come in for longer periods of time. Thus, methadone units may have more ongoing connections to their local communities through their clientele and be more aware of prevention needs.

Another unit-level factor expected to increase the likelihood of new prevention and outreach services was participation by volunteers, whose presence could both reflect and enhance community orientation [28]. Finally, larger and older units may be more likely to add prevention and outreach services because they have more slack resources to pursue public service [29] and more established community ties, although a previous study in substance abuse treatment did not find age of unit to be associated with more innovative treatment methods [30].

Predictions about unit-level factors may be summarized as follows:

H5. For-profit units will be less likely than non-profit units to add prevention and outreach services.

H6. Units providing methadone maintenance services will be more likely than other units to provide prevention and outreach services.

H7. The number of volunteer hours reported by units will be positively associated with addition of prevention and outreach services.

H8. Larger units will be more likely to add prevention and outreach services.

H9. Older units will be more likely to add prevention and outreach services.

### Predictions about unit leadership factors

Organizations may face compelling environmental forces and have distinctive collective features, but they are also managed by individuals. The current study therefore also sought to incorporate the attributes of unit leaders that might affect decisions about prevention and outreach services. Previous research indicates that individuals' licensing has less of an effect than their university-based education [30,31]. Perhaps there is a greater exposure to distinct treatment philosophies in university programs. People may also tend to earn degrees during a more impressionable phase of their professional socialization than non-university based training. Whichever the reasons, this implies that formal education, but not licensing, would predict additions of prevention-related outreach as well. Because service decisions may emerge from either the top administrator or front line supervisors, these associations were tested for both unit directors and clinical supervisors.

This leads to the last hypothesis:

H10. Unit-level leaders' advanced education, but not licensing, will be associated with increased likelihood of adding prevention and outreach services.

Thus, previous theory and evidence suggest that factors at a variety of levels might affect the likelihood that substance abuse treatment units would add prevention and outreach services. Given the prevalence of at least minimal such programming, however, the question driving the current inquiry was: What factors make it more likely that units will add *new *community prevention, education, and outreach services? These analyses were conducted in the context of a longitudinal survey of outpatient substance abuse treatment units throughout the United States.

## Methods

### Data

The data used for this study were from a national sample of outpatient substance abuse treatment units surveyed in 1995 and again in 1999/2000 as part of the National Drug Abuse Treatment System Survey [32]. The National Drug Abuse Treatment System Survey is a longitudinal program of research into the organizational structure, operating characteristics, and treatment modalities of outpatient substance abuse treatment units in the United States [33], defined in this survey as physical facilities devoting at least 50% of their resources to treating individuals with substance abuse problems (including alcohol and other drugs) on a non-residential basis. As incorporated in these analyses, a treatment unit could be either self-standing or part of a larger organization, such as a mental health center or hospital. The sampling frame is the Institute for Social Research's well-maintained list of the nation's outpatient substance abuse treatment units [32,34]. The University of Michigan's institutional review board has approved all processes involved in this research program. This survey has been conducted again after 2000, but due to respondent burden concerns did not include questions about new services. Thus, the data used here are the most recent available.

Sample stratification by public/private status, treatment modality (methadone or non-methadone) and organizational affiliations yielded generality to all major types of outpatient substance abuse treatment units in the US in 1995, with the exception of hospital-based units; these were excluded because none added prevention or outreach services during the study period (thus, hospital affiliation perfectly predicted the outcome and could not be retained in the multiple regression model). Research staff pilot tested instruments and built probes and follow-up questions into the interview protocols to enhance validity and reliability of the data. Survey staff interviewed the director and clinical supervisor of each participating unit separately, asking each about his or her areas of greatest expertise (for instance, they asked directors about new service additions and asked clinical supervisors about current services).

After screening and non-response, the total number of organizations completing interviews in 1995 was 618, for a combined response rate of 86%. A similar sampling process in 1999/2000 resulted in 745 organizations completing interviews for a response rate of 89%. 489 organizations were present in both 1995 and 1999/2000. After listwise deletion, the final sample size was 450. Because only those organizations that completed surveys in both 1995 and 1999/2000 were included, the analysis sample is not nationally representative to the extent that changes in the population of treatment providers that occurred after 1995 are not reflected in the data. However, the lagged panel design allows stronger attribution about causal relationships.

### Measures

The dependent variable identifies those units that added general or AIDS-related education, prevention, or outreach to the community between 1995 and 1999/2000. Each organization's director provided this information in response to forced choice prompts specifically concerning additions of : (1) "community education prevention, and outreach," classified as substance-abuse related (n = 19); (2) "community: AIDS education, information," classified as substance-abuse related (n = 3); (3) "community service: education, prevention, or outreach," classified as non-substance abuse related (n = 18); and (4) "community service: AIDS education, information," classified as non- substance abuse related (n = 5). For purposes of the current analysis, a composite variable for community prevention and outreach = 1 if the director responded affirmatively concerning the addition of any one of these four options. Conceptually, this made it possible to examine a set of prevention and outreach activities addressing highly inter-related risky behaviors. Empirically, because additions of HIV-AIDS prevention activities were so rare, it was necessary to combine responses about that domain with a broader response category to have sufficient statistical power to detect associations with possible predictors.

The initial multiple regression model included two measures of the unit's local market (per capita income and overall perceived competition [alpha = .83 for a five item scale]); three measures reflecting ties to other organizations (the presence of managed care contracts, being based in a hospital, and being based in a mental health center); and six organizational attributes (private for-profit or public ownership versus private not-for-profit status; methadone status; number of volunteer hours; number of clients; and facility age). All the units in the sample already provided some level of community education, outreach, or prevention services in 1995. Thus, although previous related programming would have been a relevant predictor, because there was no variation it was excluded from the model. Finally, four measures reflected characteristics of the unit's leadership (substance abuse treatment licenses held by director and clinical supervisor, director graduate degree, and clinical supervisor graduate degree). Each measure is described in greater detail in Table 1.

**Table 1:** Description of study measures

**Measure**	**Description**	**Source/Response Options**
New education, prevention or outreach services	Units adding general or AIDS education, prevention, or community outreach services between 1995 and 1999/2000	Director (Yes = 1)
Per capita income	Per capita income of county where unit is located	Area Resource File
Competition	Degree of competition as perceived by the director (α = .83)	Director
Managed care contract(s)	Unit has one or managed care arrangements	Director (Yes = 1)
Hospital affiliation	Unit is part of a hospital	Director (Yes = 1, referent group includes freestanding units or those with "other" affiliation)
Mental health center affiliation	Unit is part of a mental health center	Director (Yes = 1, referent group includes freestanding units or those with "other" affiliation)
Private for-profit ownership	Unit ownership or control status is private, for-profit	Director (Yes = 1, referent group is private nonprofit units)
Public ownership	Unit ownership or control status is public	Director (Yes = 1, referent group private for-profit units)
Methadone	Unit provides methadone treatment services to clients	Director (Yes = 1)
Previous community education or outreach services	Unit provided community education, outreach, or prevention services in 1995	Clinical Supervisor (Yes = 1)
Volunteer hours (log transformed)	Number of volunteer hours in the most recent complete fiscal year	Clinical Supervisor
Number of clients (log transformed)	Total number of clients in most recent complete fiscal year	Director
Unit age	Years of unit substance abuse treatment operations	Director
Director substance abuse treatment license	Director holds at least one professional license	Director (Yes = 1)
Supervisor substance abuse treatment license	Clinical supervisor holds at least one professional license	Clinical supervisor (Yes = 1)
Director graduate degree	Director has a degree beyond a bachelors	Director (Yes = 1)
Supervisor graduate degree	Director has a degree beyond a bachelors	Clinical supervisor (Yes = 1)

### Analyses

A multiple logistic model estimated associations between each predictive factor and the likelihood of adding general or AIDS education, prevention or outreach services to the community between 1995 and 1999/2000. Stratification variables (methadone status, hospital affiliation, mental health center affiliation, and for-profit ownership) accounted for probability of entry into the study and for non-response [32,34].

## Results

### Descriptive results

Table 2 lists summary descriptive statistics for new prevention, education, and outreach services between 1995 and 1999/2000 as well as predictors from 1995. In 1999/2000 7% of facility directors reported having added new prevention-related services during the previous 5 years.

**Table 2:** Descriptive Statistics

**Variable**	**Mean**	**Minimum**	**Maximum**	**SD**
New education, prevention, or outreach services initiated	0.07	0.00	1.00	0.26
Per capita income (in $000)	24.12	11.49	58.10	7.86
Competition	25.09	5.00	46.00	8.42
Managed care (2)	0.71	0.00	1.00	0.45
Hospital-based (3)	0.15	0.00	1.00	0.36
Mental health center-based (3)	0.23	0.00	1.00	0.42
Private for-profit (4)	0.12	0.00	1.00	0.32
Public (4)	0.27	0.00	1.00	0.44
Methadone facility (2)	0.24	0.00	1.00	0.42
Prior education/outreach (2)	1.00	1.00	1.00	0.00
Volunteer hours (log transformed)	2.32	0.00	9.21	3.02
Number of clients (log transformed)	6.08	3.26	9.21	1.02
Unit age	16.54	0.00	40.00	8.00
Director license (2)	0.51	0.00	1.00	0.50
Supervisor license (2)	0.59	0.00	1.00	0.49
Director graduate degree (2)	0.70	0.00	1.00	0.46
Supervisor graduate degree (2)	0.69	0.00	1.00	0.46

Notes:

(1) N = 450 treatment units

(2) Yes = 1

(3) Yes = 1; other or no affiliation is referent category

(4) Yes = 1; private not-for-profit is referent category

### Environmental factors

Results of the final regression model are shown in Table 3. All else being equal, for every $1,000 increase in county per capita income, treatment units had 5% higher odds of adding prevention and outreach services, relative to not adding such services. Given an $8,900 difference between per capita incomes at the 25^th ^and 75^th ^percentiles, respectively ($19,170 vs. $27,070) during the study period, this translated into 44% greater odds of prevention-related service additions for units in counties at the higher level of income. Relationships with both payers and parent organizations were associated with prevention-related education and outreach services. Units with managed care contracts had only 42% of the odds of adding these services as units without managed care contracts. No hospital-affiliated treatment units added prevention or outreach services during the study period. This factor was thus omitted from the final model due to perfect prediction. In contrast, units affiliated with mental health centers were more likely as other units to add these services.

**Table 3:** Results of Logistic Regression: Factors Predicting Substance Abuse Treatment Units' Additions of New Outreach and Prevention Services (1)

	Odds Ratio	Std Err	P > Z	95% Confidence Interval
**Environmental**				

Per capita income	1.050	0.024	0.032 *	1.004 – 1.099
Competition	0.961	0.024	0.112	0.916 – 1.009
Managed care (2)	0.421	0.178	0.041 *	0.183 – 0.965
Mental health center affiliation (3)	3.131	1.430	0.012 *	1.279 – 7.664
				
**Unit-level**				
Private for-profit ownership (4)	0.280	0.303	0.240	0.034 – 2.335
Public ownership (4)	0.477	0.237	0.137	0.180 – 1.265
Methadone (2)	0.179	0.129	0.017 *	0.044 – 0.733
Volunteer hours	1.040	0.070	0.557	0.912 – 1.186
Number of clients	0.767	0.161	0.206	0.508 – 1.158
Unit age	1.015	0.026	0.571	0.964 – 1.068
				
**Unit leadership**				
Sub abuse treatment license (director) (2)	0.895	0.430	0.818	0.349 – 2.295
Sub abuse treatment license (supervisor) (2)	0.830	0.401	0.700	0.322 – 2.138
Director advanced education (2)	0.347	0.188	0.051	0.120 – 1.006
Supervisor advanced education (2)	4.476	2.845	0.018 *	1.288 – 15.559
Pseudo R-squared statistic: 0.158				

Notes:

(1) N = 450 treatment units

(2) Yes = 1

(3) Yes = 1; other or no affiliation is referent category

(4) Yes = 1; private not-for-profit is referent category

* p < 0.05

### Unit-level factors

Two of the six unit-level factors examined were associated with the likelihood of adding prevention-related education and outreach. The type of facility ownership was unrelated to the service additions examined. Contrary to expectations, methadone units had on average only about one-fifth the odds of adding prevention-related education and outreach as non-methadone units. The number of volunteer hours was unrelated to likelihood of adding prevention and outreach services.

### Unit leadership

Previous research had suggested that leaders' licences would be unrelated to the likelihood of adding new services, but that university degrees would predict these additions. In keeping with the first expectation, there was no association between director or supervisor substance abuse treatment licenses and additions of prevention and outreach. Although, contrary to second prediction, units whose directors had graduate degrees were on average had only about a third the odds of other units of adding new prevention and outreach, this was not statistically significant at alpha = 0.05. In keeping with this latter prediction, however, units whose clinical supervisors had graduate degrees had odds over four times greater than those of otherwise comparable units to add these services.

## Discussion

This investigation began with an interest in what factors might prompt substance abuse treatment units to add prevention and outreach services. Such facilities are logical players in the prevention of risky behaviors. In keeping with an ecological perspective on organizational strategy, the overall expectation was that factors across multiple levels would affect the likelihood that units would offer new outreach and prevention services. Findings supported this expectation, with support for one or more predictors for each level (environmental, unit level, and unit leadership).

As predicted, findings indicate that substance abuse treatment units in more affluent communities were more likely to add prevention-related education and outreach during the study period. Risky substance use and sexual behaviors know no class boundaries. There is arguably no community that does not need prevention-related education. However, behavioral health problems often affect low income areas even more than high income areas [35-38]. Thus, it is unfortunate, if unsurprising, to find an association between community resource abundance and service additions in this vital area. Those funding prevention-related outreach and education may want to specifically focus on lower income populations.

The fact that managed care contracts were negatively associated with the likelihood of adding prevention and outreach services may indicate that this type of financing discourages prevention-related programs. This suggests that regions with very high managed care penetration may be particularly likely to have inadequate prevention and outreach, although future research may reveal that different types of managed care arrangements have varying effects. To the extent that substance abuse treatment is publicly funded, however, legislators may be able to change this effect by requiring that payment for health education and outreach be included in managed care contracts. For instance, the majority of states now use managed care to pay for Medicaid-funded substance abuse treatment [24]. Medicaid managed care contracts may therefore be a logical mechanism for supporting increased prevention and outreach programming. Altering Medicaid managed care contracts would also address the recommendation made above to focus prevention-related funding on lower income populations.

Institutional theory had suggested that substance abuse treatment units based in hospitals would be less likely to add prevention and outreach because such additions would be inconsistent with hospitals' remediative and individual patient orientation. The same perspective suggested that mental health center-based units would be more likely to add new prevention and outreach because prevention has traditionally been salient in mental health care. Findings from the current study supported these predictions. No hospital-based units added prevention or outreach services during the study period. In contrast, mental health center-based units were more likely than other facilities to add these services. These findings are congruent institutional theory's emphasis on culture and belief systems. Another possibility, however, is that some hospitals are not set up to receive grants supporting prevention and outreach. To the extent that such financial structures rather than hospital cultures are the main barrier, units may be able to increase prevention and outreach if funds are earmarked through managed care payments.

Hospital leaders may benefit their communities by considering local needs for prevention services and looking for new ways to address those. If institutional theory does apply, this case will need to be made in a manner consistent with the more business oriented culture of hospitals. A business case could be made for prevention and outreach on the basis that they improve the hospital's visibility in its local market and/or because philanthropic or public funding could support their costs.

Previous evidence indicated that methadone units would be more likely to add prevention-related services. Instead, in this sample they were less likely than other types of units to add these services. The a priori logic was that methadone units' higher functioning, long-term clients might foster greater awareness of local prevention needs. Although data available for this study do permit in-depth examination of this issue, methadone units differ from other types of substance abuse treatment facilities in a range of factors that may affect prevention programming. Methadone units have distinct types of services, clients, payment mechanisms [39], and legal oversight [40]. It is possible, for instance, that their revenue streams are restricted in ways that preclude prevention-related program additions.

The facts that all units in the sample were already offering some kind of community services, yet only 7% added any new services in this area during the study period, are themselves worth noting. It is possible that substance abuse treatment units feel a normative pressure to do something about community outreach – but not too much. Future research should investigate the extent and impact of community outreach and education services by a variety of health care providers as well as what factors prompt varying levels of such services.

Regardless of environmental and organizational factors, leaders exercise discretion about what services to offer. This is particularly applicable to public goods that typically enhance mission accomplishment but may undermine financial margin. Directors may decide to launch new prevention and outreach initiatives; supervisors may tacitly expand unit strategy by sending staff to community health fairs and similar events to promote healthy behaviors. This study acknowledged the possibility that such service additions could therefore reflect both director and supervisor influence.

As expected on the basis of previous literature, facility director and supervisor licensing in substance abuse treatment were unrelated to additions of new prevention-related services during the study period. The mixed effects of graduate degrees in the current sample suggest that facility leadership's university education may affect their inclination to provide public goods. In the current sample, the majority of both directors and clinical supervisors had graduate degrees in counselling, education, or social work (44% of directors and 48% of supervisors) and the most common single type of degree was in psychology (35% of directors and 38% of supervisors). However, unsurprisingly, directors were 50% more likely to have administrative degrees than supervisors (12% versus 8%) and twice as likely to have earned those degrees in business administrative programs (8% versus 4%). Supervisors were slightly more likely than directors to have graduate degrees in counselling (19% versus 16%) and psychology (38% versus 35%).

It is possible that business administration programs emphasize financial efficiency norms while other types of graduate degree programs (e.g., counselling and psychology) promote a more holistic perspective on organizational performance including more emphasis on societal goals. Public funding may be used to encourage graduate education that supports a more holistic view of agencies within their broader social contexts. This may become increasingly important if more leaders in health and human service earn business administration degrees.

### Limitations and future research

The current study had limitations that suggest the need for complementary research on this under-examined topic. Empirically, the large number of associations tested makes it very likely that at least one of the findings from this study would not be replicated in another similar sample (i.e., was a Type I error). This suggests treating results from these analyses with caution and looking at patterns across studies before drawing strong conclusions. In general, benefits of breadth of necessity occurred at the expense of depth (due primarily to concerns about respondent burden from director and supervisor interviews). Future research should probe more fully the potential leverage points identified in this study. For instance, what kinds of payment structures, if any, are positively associated with more additions of prevention and outreach services? What kinds of graduate training enhancements affect attitudes toward public goods such as prevention, and do such attitudes actually translate into strategic behavior? Both questions suggest pre-post experimental studies with longitudinal follow-up. For instance, some states could add a prevention component to Medicaid managed care payments for substance abuse treatment (potentially with varying levels, to identify the thresholds at which impact increases), allowing for comparison with units in non-intervention states over time. Similarly, a foundation could enroll management graduate programs in a matched study design tracing effects of specific modules on student attitudes. It could even be feasible to track differences in subsequent actions of program graduates using self-report data.

These or other studies might also address another limitation of the current investigation, which was the dichotomous nature of both some key predictors (i.e., the pre-existence of prevention-related services and presence of managed care contracts) and the outcome measure in the current study. Better measures of existing commitment to prevention services could include the number of staff members and annual expenditures devoted to these activities. Measures of new prevention and outreach should include not only levels of resource commitment but also whether or not they are truly evidence based. Use of such measures, combined with longitudinal designs, could facilitate identification of causal sequences that would inform more operationally specific policy recommendations. The authors do not, however, believe that such investigations would yield policy recommendations differing from those from this study's. For instance, it is possible that units that are more reliant on managed care have lower pre-existing commitments to prevention and outreach than other units, but it is not likely that the association found here between managed care and new prevention-related services was causally attributable to the (unmeasured) level of current services. Concerning the other major policy lever identified in this study, leadership graduate education, it is not plausible that levels of prevention programming affected leaders' education. Thus, more refined measurement is likely to complement rather than contradict conclusions presented here.

## Conclusion

The personal, social, and economic effects of substance abuse are staggering. There are compelling moral and economic reasons to make a far greater public investment in prevention-related education and outreach than currently exists. At this point, however, investment lags far behind rhetoric, and current US fiscal trends bode badly for the immediate future of this field. It is thus important to understand what encourages health care providers to add such vital services in the absence of adequate reimbursement. The current analyses indicate that there are leverage points for encouragement of additional prevention and outreach services. Most notably, the structure of managed care contracts and some types of graduate degree programs for facility leadership may encourage more such services. In all of these cases, relatively modest investments of public funds may facilitate services with great importance to individual and societal health.

## Competing interests

The author(s) declare that they have no competing interests.

## Authors' contributions

RW conceived and conducted the analyses and drafted the background section. CHL provided input into the analyses and drafted the methods and results sections. TD designed the study from which the data derive, provided input into the analyses, and contributed to the discussion section. All authors read and approved the final manuscript.
